# Molecular and cellular modulators for multisensory integration in *C*. *elegans*

**DOI:** 10.1371/journal.pgen.1007706

**Published:** 2019-03-08

**Authors:** Gareth Harris, Taihong Wu, Gaia Linfield, Myung-Kyu Choi, He Liu, Yun Zhang

**Affiliations:** Department of Organismic and Evolutionary Biology, Center for Brain Sciences, Harvard University, Cambridge, MA, United States of America; University of California San Francisco, UNITED STATES

## Abstract

In the natural environment, animals often encounter multiple sensory cues that are simultaneously present. The nervous system integrates the relevant sensory information to generate behavioral responses that have adaptive values. However, the neuronal basis and the modulators that regulate integrated behavioral response to multiple sensory cues are not well defined. Here, we address this question using a behavioral decision in *C*. *elegans* when the animal is presented with an attractive food source together with a repulsive odorant. We identify specific sensory neurons, interneurons and neuromodulators that orchestrate the decision-making process, suggesting that various states and contexts may modulate the multisensory integration. Among these modulators, we characterize a new function of a conserved TGF-β pathway that regulates the integrated decision by inhibiting the signaling from a set of central neurons. Interestingly, we find that a common set of modulators, including the TGF-β pathway, regulate the integrated response to the pairing of different foods and repellents. Together, our results provide mechanistic insights into the modulatory signals regulating multisensory integration.

## Author summary

The present study characterizes the modulation of a behavioral decision in *C*. *elegans* when the worm is presented with a food lawn that is paired with a repulsive smell. We show that multiple specific sensory neurons and interneurons play roles in making the decision. We also identify several modulatory molecules that are essential for the integrated decision when the animal faces a choice between the cues of opposing valence. We further show that many of these factors, which often represent different states and contexts, are common for behavioral decisions that integrate sensory information from different types of foods and repellents. Overall, our results reveal the molecular and cellular basis for integration of simultaneously present attractive and repulsive cues to fine-tune decision-making.

## Introduction

An environment is often represented by numerous sensory cues. In order to better survive, an animal often needs to detect and process simultaneously present sensory cues to make a behavioral decision [1–8]. Because integrating multiple sensory cues generates a more accurate evaluation of the environment, it provides important adaptive values. Multisensory integration is widely observed in both vertebrate and invertebrate animals. Previous studies using behavioral and psychophysical approaches show that humans and other organisms can integrate an array of sensory stimuli to generate decisions in every-day life [9–11]. One common characteristic of multisensory behavioral responses and decision-making processes is their ability to be modulated by various internal states and contexts, including arousal, sleepiness versus wakefulness, the motivational or nutritional state of the organism, or the level of the reward paired with the stimuli. Neurotransmitters, such as dopamine, serotonin, glutamate, and neuropeptides, mediate many of these neurological effects on decision-making. In addition, patients of several neurological diseases, including autism spectrum disorder and schizophrenia share deficits associated with sensory processing or decision-making when encountering multiple sensory stimuli [3, 4, 12–20]. Together, these studies reveal multisensory integration as a common neuronal and behavioral process modulated by multiple contexts across the animal kingdom.

Despite the importance of multisensory integration in animal behavior, our understanding of the underlying mechanisms remains preliminary. The nematode *C*. *elegans* provides an opportunity to address the question. *C*. *elegans* feeds on bacteria. A bacteria lawn provides various types of sensory information, including olfactory, gustatory, mechanical, and gaseous cues. The small nervous system (302 neurons) of *C*. *elegans* hermaphrodite generates sensorimotor responses to these modalities [21–32] and many of the responses can be shaped by external and internal contexts that modulate neural activities [4, 33–37]. The *C*. *elegans* genome encodes the homologues of about 50% of the molecules expressed in the mammalian brains [38], which in combination with a well-defined wiring diagram of the nervous system [39] allows characterization of the molecular and circuit basis for multisensory integration during decision-making.

Here, we show that *C*. *elegans* integrates the information from an attractive food lawn and a simultaneously present repellent to generate a decision on leaving. We show that the decision to leave the lawn depends on the attractiveness of the lawn and the concentration of the repellent. We identify specific neurons and modulatory molecules that promote or suppress the food-repellant integration underlying the lawn-leaving decision. We further demonstrate that several modulatory molecules and neurons act as common modulators to regulate integrated decisions on different foods paired with different repellents. These findings identify conserved neuronal signals that modulate multisensory processing during decision-making and reveal a modulatory basis for multisensory integration.

## Results

### *C*. *elegans* integrates multiple sensory cues to generate a behavioral decision

**Fig 1:**
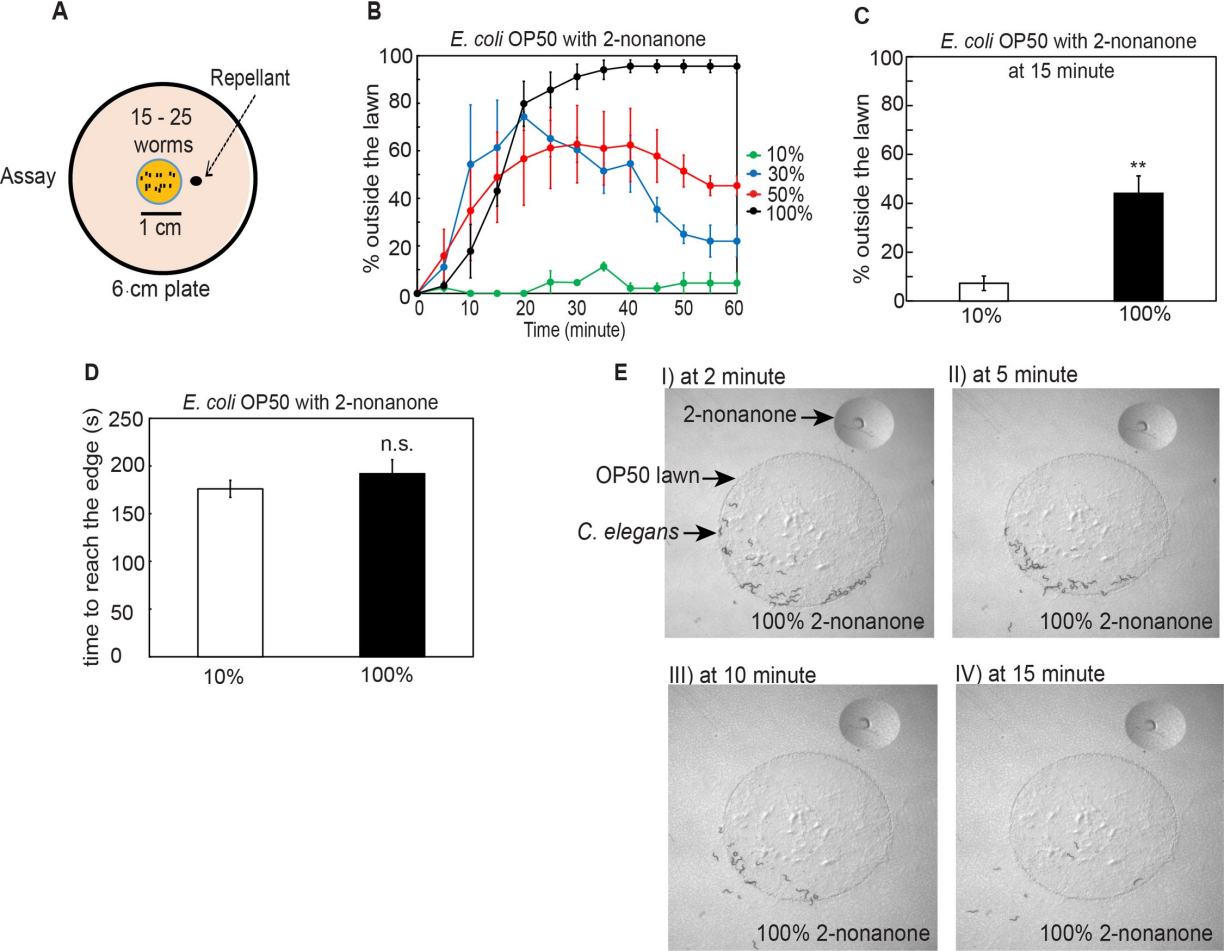
*C*. *elegans* performs multisensory integration to leave food paired with a repulsive odorant 2-nonanone. **(A)** A schematic of 2-nonanone-dependent food leaving assay.**(B)** The time course for worms leaving an OP50 lawn that is paired with 2-nonanone of different concentrations over 60 minutes, n = 2 assays for 10% and n = 3 assays each for 30%, 50% and 100%. **(C)** More worms leave the OP50 food lawn paired with 100% 2-nonanone (n = 4 assays) than the OP50 lawn paired with 10% 2-nonanone (n = 5 assays). Bar graph represents the percentage of worms outside the lawn 15 minutes after the assay starts. **(D)** The time taken for worms to reach the edge of the OP50 food lawn when the lawn is paired with either 10% or 100% 2-nonanone, n = 2 assays each. **(E) I–IV,** Sample images of wild-type animals leaving an OP50 lawn that is paired with 100% 2-nonanone at different time points of the 60-minute assay. For **B-D**, Mean ± SEM, Student’s *t* test, ** p ≤ 0.01, n.s., not significant.

To establish a behavioral assay for multisensory integration in *C*. *elegans*, we presented a repulsive odorant, 2-nonanone, to the animals on a small lawn of the *E*. *coli* strain OP50 (Fig 1A and Experimental Procedures) and assessed the decision of the animals to stay on or leave the lawn over time. Because the OP50 lawn serves as a food source for the worm, under the standard condition *C*. *elegans* stays on the lawn and leaves only at a low frequency [40, 41]. Meanwhile, 2-nonanone strongly repels *C*. *elegans* at concentrations ranging from 10% to 100%. The olfactory sensory neurons AWB detect and mediate the avoidance of 2-nonanone [42–44]. We first presented a drop of 10% 2-nonanone close to the edge of an OP50 lawn, on which 15–25 young adults acclimatized for one hour (Fig 1A). We found that within a few minutes the animals migrated to the side of the bacterial lawn away from 2-nonanone, stayed on the edge of the lawn before dispersing throughout the lawn without leaving (Fig 1B). This result indicates that *C*. *elegans* is able to detect and avoid 10% 2-nonanone even on the food lawn, but the repulsion is not strong enough to suppress the retention of the worm by the food lawn. In contrast, when we presented a drop of higher concentration of 2-nonanone to the worms in the same configuration, the worms migrated to the side of the lawn, started to leave the food lawn in a few minutes, and continued to migrate to the edge of the plate away from the repellent within the one-hour time window of the assay (Fig 1B and 1C and S1 Movie). The food-leaving behavior was robustly evoked with 100% 2-nonanone (Fig 1B–1E), under which condition a significant number of worms already left the lawn after 2-nonanone was presented to the worms for 15 minutes (Fig 1C). In addition, it took a similar amount of time for the worms to reach the edge of the lawn that was paired with either 10% or 100% 2-nonanone (Fig 1D). These results show that *C*. *elegans* integrates the attraction of a food lawn with the repulsion of 2-nonanone to generate a behavioral decision and that increasing concentration of 2-nonanone enhances lawn leaving (Fig 1B and 1C). These findings are consistent with the general rule that governs multisensory integration, where increasing the reliability of a sensory cue, such as increasing the concentration of 2-nonanone, strengthens the weight of the cue in integration [45]. To characterize the regulatory mechanisms underlying multisensory integration, we used 100% 2-nonanone as the repellent for the rest of the study unless otherwise described. We quantified the percentage of the worm outside the OP50 lawn 15 minutes after the assay began unless otherwise described, because it was an early time point when wild type started to show a robust leaving decision.

### Sensory neurons that regulate multisensory integration

**Fig 2:**
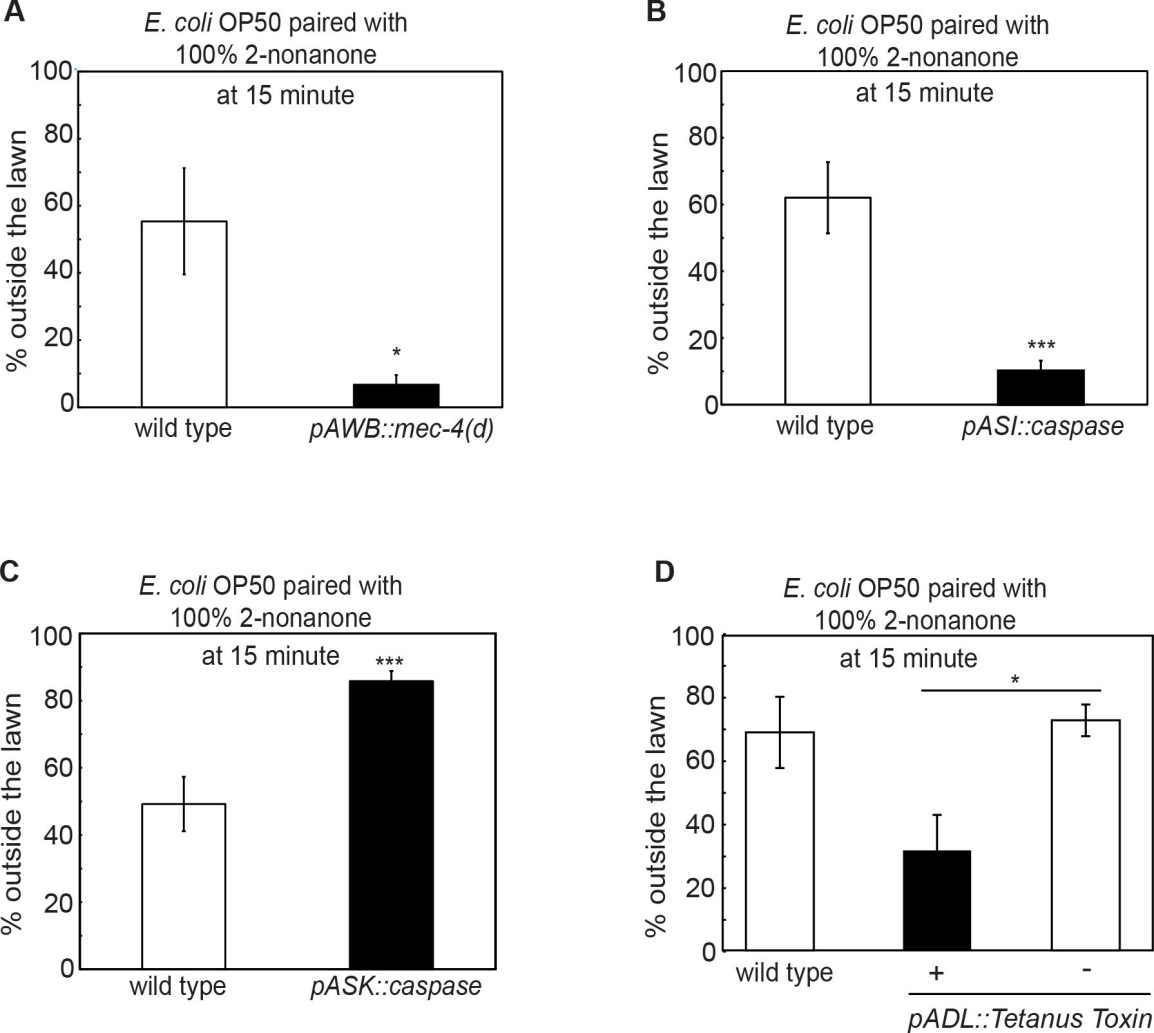
Several sensory neurons modulate 2-nonanone-dependent food leaving. **(A-D)** The transgenic animals that either lack the functional AWB sensory neurons by selectively expressing the gain-of-function isoform of an amiloride-sensitive sodium channel MEC-4 (**A**, *pAWB*::*mec-4(d)*, n = 5 assays each) or lack the ASI sensory neurons by expressing a cell death promoting molecule caspase (**B**, *pASI*::*caspase*, n = 7 assays for wild type and 8 assays for the transgenic animals) or are defective in the synaptic transmission of the ADL sensory neurons by expressing the tetanus toxin (**D,** *pADL*::*TeTx*, n = 5 assays for wild type, 4 assays for the transgenic animals, and 3 assays for non-transgenic siblings) display a delayed decision to leave the OP50 lawn paired with 100% 2-nonanone; while the transgenic animals that express caspase in the ASK sensory neurons (**C,** *pASK*::*caspase*, n = 9 assays each) display a faster decision to leave. Each bar graph reports the average percentage of worms outside the lawn 15 minutes after the assay starts. Mean ± SEM, Student’s *t-t*est, * p ≤ 0.05, ***p ≤ 0.001.

To characterize how the nervous system regulates the integrated response to the attractive OP50 lawn and the repulsive odorant 2-nonanone, we first probed the amphidal sensory neurons AWB, which mediate avoidance of 2-nonanone via the function of the cGMP-gated channel subunit *tax-2* [42]. Exposure to 2-nonanone suppresses the intracellular calcium transients of AWB [43, 44]. Consistently, we found that the transgenic animals that selectively expressed a hyperactive form of an amiloride-sensitive sodium channel MEC-4 that generated necrosis of AWB [42, 46] did not leave the OP50 lawn when 2-nonanone was present (Fig 2A) and that many of the worms remained diffusely distributed on the food lawn by the end of the assay. These transgenic animals were defective in avoiding 2-nonanone in the standard chemotaxis assay (Table 1 and S1 Fig), consistent with previous findings [42]. AWB-killed animals also spent more time to reach the edge of the OP50 lawn when 2-nonanone was present (Table 2), consistent with the role of AWB in mediating the avoidance of 2-nonanone. Meanwhile, the transgenic animals with genetically killed AWB stayed on OP50 lawn similarly as wild type when 2-nonanone was not present (Table 3). Together, these results show that AWB regulate the integrated response by mediating the response to the unisensory repellent 2-nonanone.

**Table 1:** 2-nonanone avoidance assay. Wild type, mutants and transgenic animals are examined for avoiding 100% 2-nonanone as previously described (Troemel et al., 1997 and S1 Fig). Avoidance Index was calculated as described in S1 Fig. The avoidance in each genotype is represented by the average avoidance index of individual assays. n = 2–4 assays each genotype, 75–100 animals tested in each assay, mutants and transgenic animals are compared with wild-type animals or the non-transgenic siblings tested on the same days with student’s *t* test, Mean ± SEM.

Strain	Strain information	Avoidance Index Mean ± SEM	p value
Wild type		-0.97 ± 0	
QS4	ASK-killed	-0.959 ± 0.007	P > 0.05
Wild type		-0.955 ± 0.0144	
PY7505	ASI-killed	-0.921 ± 0.0138	P > 0.05
Wild type		-0.987 ± 0.012	
CX3831	AWB-killed	-0.482 ± 0.106	P = 0.041
Wild type		-0.965 ± 0.005	
FX02984 *nlp-7(tm2984)*	Neuropeptide NLP-7	-0.992 ± 0.007	P > 0.05
*ZC1552* *Pglr-1*::*TeTx ntg*		-0.845 ± 0.035	
*ZC1552* *Pglr-1*::*TeTx tg*	Blocking synaptic release from command interneurons & some motor neurons	-0.875 ± 0.019	P > 0.05
Wild type		-0.984 ± 0.004	
DR40 *daf-1(m40)*	Type I TGF-β receptor	-0.915 ± 0.025	P > 0.05
Wild type		-0.96 ± 0.020	
ZC2393 *Pttx-3*::*twk-18(gf)*	Expressing *twk-18(gf)* in AIY	-0.915 ± 0.005	P > 0.05
Wild type		-0.825 ± 0.075	
ZC1451 *Pnmr-1*::*TeTx*	Blocking synaptic release from command interneurons	-0.879 ± 0.0003	P > 0.05
Wild type		-0.972 ± 0.002	
*ZC1952* *Pttx-3*::*TeTx*	Blocking synaptic release from AIY interneurons	-0.925 ± 0.020	P > 0.05
Wild type		-0.933 ± 0.003	
OH8 *ttx-3(mg158)*	Disrupting development of AIY interneurons	-0.966 ± 0.013	P > 0.05
Wild type		-0.98 ± 0.019	
VC48 *kpc-1(gk8)*	Reduction of peptides processed by KPC-1	-0.81 ± 0.005	P = 0.014
Wild type		-0.785 ± 0.015	
CX12330 *Psre-1*::*TeTx*	Blocking synaptic release from ADL	-0.963 ± 0.003	P = 0.007
Wild type		-0.87 ± 0.030	
CB1372 *daf-7(e1372)*	TGF-β ligand	-0.805 ± 0.055	P ≥ 0.4078
Wild type		-0.90 ± 0	
RB2302 *daf-7(ok3125)*	TGF-β ligand	-0.83 ± 0.030	P ≥ 0.144

**Table 2:** Time to reach the edge of the food lawn during multisensory integration. Wild type, mutants and transgenic animals are examined for the time taken to reach the edge of the food lawn away from the repellent. The average time taken for 90% of the worms in one assay to reach the edge of a *E*. *coli* OP50 food lawn during exposure to 100% 2-nonanone is presented for each genotype (Experimental Procedures and S1 Fig). n = 2–4 assays for each genotype, 20–25 animals tested in each assay, mutants or transgenic animals are compared with wild-type animals or non-transgenic siblings tested on the same days with student’s *t* test, Mean ± SEM.

Strain tested	Strain information	Time (second) Mean ± SEM	p value
Wild type		203.66 ± 18.47	
QS4	ASK-killed	207.0 ± 26.53	P > 0.05
Wild type		210 ± 30.08	
PY7505	ASI-killed	264 ± 24.07	P > 0.05
N2		205.5 ± 25.57	
CX3831	AWB-killed	542.50 ± 2.50	P = 0.0057
Wild type		219.0 ± 16.66	
FX02984 *nlp-7(tm2984)*	Neuropeptide NLP-7	253.75 ± 12.57	P > 0.05
ZC1552 *Pglr-1*::*TeTx ntg*		206.26 ± 15.06	
ZC1552 *Pglr-1*::*TeTx tg*	Blocking synaptic release from command interneurons & some motor neurons	337.86 ± 40.04	P = 0.0314
Wild type		176.5 ± 33.60	
DR40 *daf-1(m40)*	Type I TGF-β receptor	210 ± 30.08	P > 0.05
Wild type		200.66 ± 19.69	
CB1372 *daf-7(e1372)*	TGF-β ligand	243 ± 24.61	P > 0.05
Wild type		215 ± 5.014	
ZC2393 *Pttx-3*::*twk-18(gf)*	Expressing *twk-18(gf)* in AIY	222.50 ± 17.552	P > 0.05
ZC1451 *Pnmr-1*::*TeTx ntg*		181.93 ± 12.19	
ZC1451 *Pnmr-1*::*TeTx tg*	Expressing *TeTx* in command interneurons	186.83 ± 17.07	P > 0.05
Wild type		190 ± 10.02	
OH8 *ttx-3(mg158)*	Disrupting development of AIY	235 ± 5.01	P > 0.05
Wild type		227.5 ± 10.53	
VC48 *kpc-1(gk8)*	Reduction of peptides processed by KPC-1	260.5 ± 8.52	P > 0.05
Wild type		205.5 ± 25.57	
MT150 *egl-3(n150)*	Reduction of peptides processed by EGL-3	245 ± 30.08	P > 0.05
Wild type		223.5 ± 0.50	
CX12330 *Psre-1*::*TeTx*	Blocking synaptic transmission from ADL	229.5 ± 3.51	P > 0.05

**Table 3:** Spontaneous food leaving from an OP50 lawn without pairing with 2-nonanone. Wild type, mutant animals and transgenic animals are examined for food leaving for 1 hour. Young adult worms are placed on an OP50 food lawn for 10 minutes and the number of worms on food lawn is counted every 5 minutes for a total assay time of 60 minutes. The percentage of worms off the food lawn at 15 minutes is reported. n = 2–4 assays for each genotype and 20–25 worms in each assay, mutants or transgenic animals are compared with wild-type animals tested in parallel with student’s *t* test, Mean ± SEM.

Strain tested	Strain information	% outside lawn Mean ± SEM	p value
Wild type		2.17 ± 2.18	
QS4	ASK-killed	9.92 ± 4.37	P > 0.05
Wild type		0 ± 0	
PY7505	ASI-killed	0 ± 0	P > 0.05
Wild type		0 ± 0	
CX3831	AWB-killed	2.17 ± 2.18	P > 0.05
Wild type		0 ± 0	
FX02984 *nlp-7(tm2984)*	Neuropeptide NLP-7	0 ± 0	P > 0.05
Wild type		9.16 ± 3.79	
DR40 *daf-1(m40)*	Type I TGF-β receptor	0 ± 0	P = 0.026
Wild type		1.13 ± 1.13	
CB1372 *daf-7(e1372)*	TGF-β ligand	0 ± 0	P > 0.05
Wild type		1.44 ± 1.45	
ZC1451 *Pnmr-1*::*TeTx*	Blocking synaptic release from command interneurons	1.51 ± 1.51	P > 0.05
Wild type		4.54 ± 4.55	
ZC1952 *Pttx-3*::*TeTx*	Blocking synaptic release from AIY interneurons	0 ± 0	P > 0.05
Wild type		4.67 ± 0.327	
MT150 *egl-3(n150)*	Reduction of peptides processed by EGL-3	2.17 ± 2.18	P > 0.05
Wild type		7.763 ± 2.77	
VC48 *kpc-1(gk8)*	Reduction of peptides processed by KPC-1	7.63 ± 2.37	P > 0.05
Wild type		7.72 ± 3.64	
CX12330 *Psre-1*::*TeTx*	Blocking synaptic release from ADL	4.34 ± 4.35	P > 0.05

Next, we sought additional sensory neurons that regulated the integrated behavioral decision. Previous studies identify several sensory neurons that respond to the smell of the *E*. *coli* strain OP50 or mediate the behavioral response to the presence or removal of food [21, 43, 47–50]. To examine the potential role of these sensory neurons in our multisensory integration paradigm, we first tested a null mutation *ky4* in *odr-7*, which encodes a putative DNA-binding nuclear receptor that specifies the function of the AWA sensory neurons [51], a null mutation *p680* in *che-1*, which encodes a zinc finger transcription factor required for the development and function of the ASE sensory neurons [52], transgenic animals that selectively express a cell-death activator EGL-1 [53] in the AQR, PQR and URX neurons or the CO_2_-sensing BAG sensory neurons [27–29, 54–56]. We also tested transgenic animals selectively expressing a cell-death inducing caspase, or *twk-18(gf)* that encodes a constitutively active form of the potassium channel TWK-18 [57], or tetanus toxin that eliminates the synaptic release [58] in the ASI, AWC, ASJ, ADL or ASK neurons [48, 54, 59–63]. We found that all except three of the tested strains were normal. The transgenic animals that contained genetically-killed ASK left the OP50 lawn significantly faster than wild type, and the transgenic animals that contained genetically-killed ASI or expressed the tetanus toxin in ADL left the OP50 lawn significantly more slowly than wild type (Fig 2B–2D and Table 4). Because the transgenic animals defective in the function of ASK or ASI or ADL are not deficient in avoiding 2-nonanone in our standard chemotaxis assay, in their ability to remain on OP50 lawn when 2-nonanone is not present, as well as in moving to the edge of the OP50 lawn with the presence of 2-nonanone (Tables 1–3), these results together indicate that the sensory neurons ASK, ASI and ADL modulate how rapidly the behavioral decision to leave the repellent-paired food lawn is made.

**Table 4:** Many signaling mutants show no phenotype in 2-nonanone-dependent food leaving. Wild type, mutant and transgenic animals are examined for leaving an *E*. *coli* OP50 food lawn paired with 100% 2-nonanone. The average percentage of worms outside the food lawn at 15 minutes is reported. Mutants or transgenic animals are compared with the wild-type control tested on the same days with student’s *t* test, n = 2–4 assays for each genotype, 20–25 animals in each assay, Mean ± SEM.

Strain	Strain information	% outside lawn Mean ± SEM	p value
Wild type		60.57 ± 20.92	
CX7102 *Pgcy-36*::*egl-1*	AQR/PQR/URX-killed	70.54 ± 27.43	p > 0.0.5
Wild type		48.80 ± 1.19	
FX02105 *nlp-24(tm2105)*	Neuropeptide NLP-24	45.77 ± 9.8	p > 0.05
Wild type		69.87 ± 3.21	
RB1902 *flp-19(ok2460)*	FMRF-amide peptides FLP-19	58.69 ± 10.90	p > 0.05
Wild type		72.50 ± 17.55	
QZ81 *ins-6(tm2416)*	ILP-like peptides INS-6	68.86 ± 3.87	p > 0.05
Wild type		36.01 ± 6.86	
QZ126 *ins-7(tm2001)*	ILP-like peptides INS-7	29.50 ± 0.98	p > 0.05
Wild type		69.27 ± 1.09	
PR680 *che-1(p680)*	Defective in ASE function	62.79 ± 8.65	p > 0.05
Wild type		70.96 ± 7.32	
FX03279 *nlp-9(tm3279)*	Neuropeptide NLP-9	54.19 ± 5.32	p > 0.05
Wild type		70.85 ± 14.90	
PY7502	AWC-killed	77.75 ± 5.41	p > 0.05
Wild type		66.24 ± 4.35	
CX4 *odr-7(ky4)*	Defective in AWA function	70.71 ± 3.54	p > 0.05
Wild type		74.51 ± 12.22	
*ASJp*::*ICE*	ASJ-killed	63.56 ± 4.62	p > 0.05
Wild type		86.09 ± 6.26	
RB1341 *nlp-1(ok1470)*	Neuropeptide like peptide NLP-1	82.06 ± 4.36	p > 0.05
Wild type		88.98 ± 1.49	
AX2051 *Pgcy-33*::*egl-1*	BAG-killed	93.18 ± 2.27	p > 0.05

### Multisensory integration requires peptidergic and the TGF-β pathways

**Fig 3:**
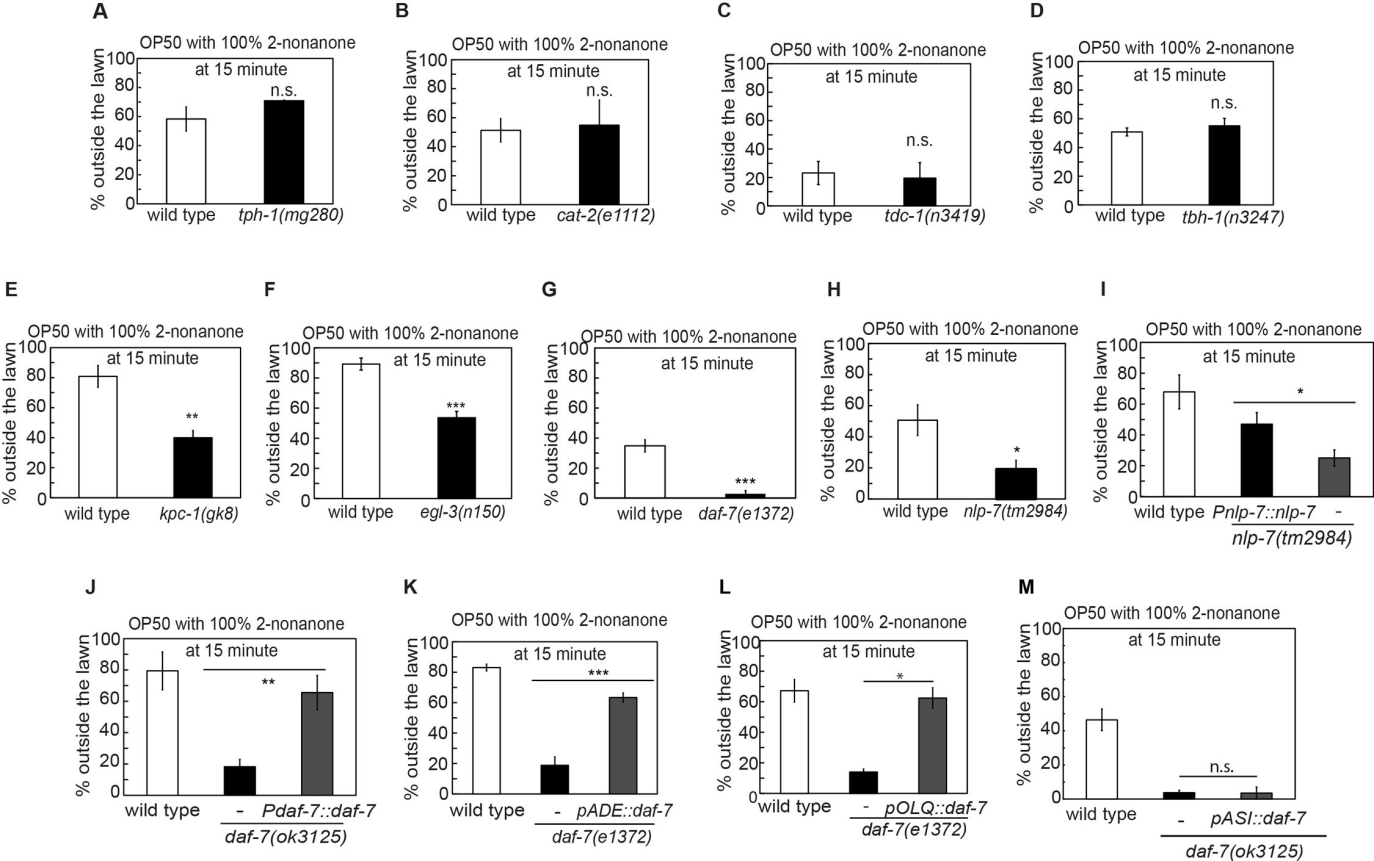
NLP-7 and TGF-β/DAF-7 modulate the decision to leave the OP50 food lawn paired with 2-nonanone. **(A-D)** The mutant animals that are defective in the biosynthesis of the neurotransmitter serotonin (**A,** *tph-1(mg280)*, n = 2 assays each), or dopamine (**B,** *cat-2(e1112)*, n = 4 assays each), or tyramine and octopamine (**C,** *tdc-1(n3419)*, n = 2 assays each), or octopamine (**D,** *tbh-1(n3247)*, n = 3 and 4 assays for wild type and *tbh-1* mutants, respectively) display a normal decision to leave the OP50 food lawn that is paired with 2-nonanone. **(E-J)** Mutations in the genes encoding the neuropeptide processing enzymes, *kpc-1* (**E,** n = 4 and 5 assays for wild type and *kpc-1* mutant, respectively), or *egl-3* (**F**, n = 4 and 6 assays for wild type and *egl-3* mutant, respectively), or in a TGF-β-encoding gene *daf-7* (**G,** n = 6 and 5 assays for wild type and *daf-7* mutant, respectively), or in a neuropeptide-encoding gene *nlp-7* (**H,** n = 7 and 8 assays for wild type and *nlp-7* mutant, respectively) generate a delayed decision to leave the OP50 food lawn paired with 2-nonanone, and expressing the genomic DNA of *nlp-7* (**I,** n = 6, 7 and 4 assays for wild type, transgenic animals and non-transgenic siblings, respectively) or the genomic DNA of *daf-7* (**J,** n = 4 assays each for wild type, transgenic animals and non-transgenic siblings) rescues the delayed food leaving phenotype of the respective mutant animals. **(K)** Expressing the wild-type *daf-7* cDNA in the sensory neurons ADE rescues the delayed decision in the *daf-7(e1372)* mutant animals, n = 4 assays each for wild type, transgenic animals and non-transgenic siblings, respectively. **(L)** Expressing the wild-type *daf-7* cDNA in the sensory neurons OLQ also rescues the delayed decision in the *daf-7(e1372)* mutant animals, n = 3 assays for wild type, 3 assays for transgenic animals and 2 assays for non-transgenic siblings, respectively. **(M)** Expressing a wild-type *daf-7* cDNA in the sensory neurons ASI does not rescue the delayed decision phenotype in the *daf-7(ok3125)* mutant animals (n = 4, 3 and 4 assays for wild type, transgenic animals and non-transgenic siblings, respectively). Each bar graph reports the average percentage of worms outside the lawn 15 minutes after the start of the assay, mutants are compared with wild-type animals and transgenic animals are compared with non-transgenic siblings using Student’s *t*-*t*est, * p<=0.05, ** p<=0.01, *** p<=0.001, n.s., not significant.

To characterize the mechanisms underlying multisensory integration of food and 2-nonanone, we examined mutants that were defective in biosynthesis of neurotransmitters. We tested effects of mutating *tph-1* that encodes tryptophan hydroxylase required for the production of serotonin [64], *cat-2* that encodes tyrosine hydroxylase needed for the synthesis of dopamine [65], *tdc-1* that encodes tyrosine decarboxylase required for the synthesis of tyramine and octopamine, or *tbh-1* that encodes tyramine beta hydroxylase required for the production of octopamine [66]. Interestingly, all of these mutants exhibited wild-type behavioral decision when they were exposed to 2-nonanone on an OP50 food lawn (Figs 3A–3D and S2). These results show that serotonin, dopamine, tyramine or octopamine are not required for 2-nonanone-dependent food leaving, although these neurotransmitters regulate many food-dependent sensorimotor responses ([31] and references therein).

Next, we examined the function of neuropeptide-encoding genes. We first found that mutations in the *kpc-1(gk8)* and *egl-3(n150)*, which disable two of the four known peptide pre-processing enzymes in *C*. *elegans* [67–69], delayed the decision to leave the food lawn paired with 2-nonanone (Fig 3E and 3F), suggesting the modulatory role of peptides or growth factors in promoting the integrated decision. Next, we screened many mutations in genes encoding peptides or growth factors. We focused on the available mutations that did not generate any gross defect in either development or locomotion and identified three mutations that significantly altered the wild-type decision. The canonical mutations, *e1372*, or a deletion, *ok3125*, in *daf-7* that encodes a TGF-β ligand that regulated development, metabolism and host-pathogen recognition [70–72], significantly delayed the decision to leave the OP50 lawn paired with 2-nonanone (Fig 3G and 3J). A deletion mutation *tm2984* in *nlp-7*, which encodes a neuropeptide-like protein that regulates stress response, egg-laying, life span and modulation of aversive responses to noxious stimuli [73–76], similarly delayed the decision to leave the lawn (Fig 3H). However, the mutations in *daf-7 or nlp-7* did not generate any detectable defect in the chemotactic response to 2-nonanone alone, or the tendency to leave the OP50 lawn when no repellent was present, or the ability to move to the edge of the lawn when 2-nonanone was present (Tables 1–3). In addition, expressing the genomic fragment containing the regulatory and coding regions of *daf-7* or *nlp-7* rescued the defect of the respective mutant animals in making the decision to leave the lawn that was paired with 2-nonanone (Fig 3I and 3J). Together, these results indicate that TGF-β/DAF-7 and NLP-7 promote the food-leaving decision when 2-nonanone is present.

### A new function of the TGF-β/DAF-7 canonical pathway in multisensory integration

**Fig 4:**
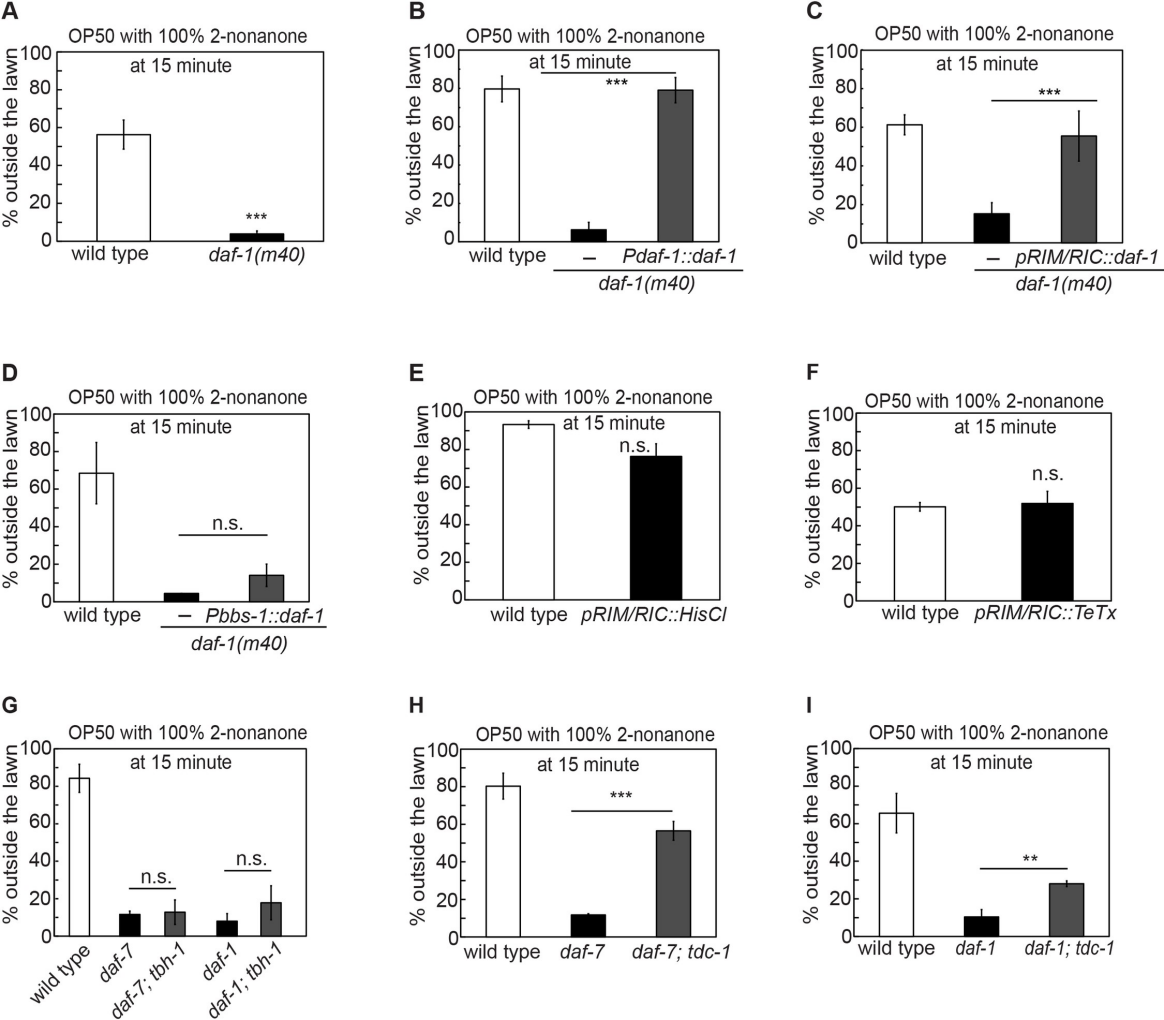
The TGF-β receptor DAF-1 acts in the RIM and RIC neurons to mediate 2-nonanone-dependent food leaving. **(A-D)** Mutating *daf-1* that encodes the type I TGF-β receptor delays the decision to leave the OP50 lawn paired with 100% 2-nonanone (**A,** n = 8 and 9 assays for wild type and *daf-1* mutant, respectively), and expressing the genomic DNA of *daf-1* (**B,** n = 6 assays each) or a wild-type *daf-1* cDNA in the RIM and RIC neurons (**C,** n = 6, 6 and 5 assays for wild type, transgenic animals and non-transgenic siblings, respectively) in the *daf-1(m40)* mutant animals rescues the delayed decision, but expressing wild-type *daf-1* in the sensory neurons (**D**, n = 3, 5 and 2 assays for wild type, transgenic animals and non-transgenic siblings, respectively) does not rescue. Mutants are compared with wild type and transgenic animals are compared with non-transgenic siblings with Student *t-*test. **(E-F)** Inhibiting the activity of the RIM and RIC neurons by selectively expressing a histamine-gated chloride channel (**E,** n = 2 and 4 assays for wild type and transgenic animals, respectively) or blocking the synaptic release from these neurons by selectively expressing the tetanus toxin (**F,** n = 2 assays each) does not alter the decision to leave the OP50 food lawn paired with 100% 2-nonanone. Transgenic animals are compared with wild type. **(G-I)** Removing octopamine signaling in the *daf-7(e1372)* or *daf-1(m40)* mutants with a mutation that disrupts biosynthesis of octopamine *tbh-1(ok1196)* does not suppress the delayed leaving from the OP50 lawn paired with 100% 2-nonanone (**G,** n = 6 assays for wild type; n = 4 assays for *daf-7* mutants; n = 2 assays for *daf-1* mutants; n = 3 assays for *daf-7;tbh-1* double mutants; n = 2 assays for *daf-1;tbh-1* double mutants), but removing the tyramine and the octopamine signals with the mutation in *tdc-1(ok914)* in either the *daf-7(e1372)* (**H,** n = 6, 5 and 4 assays for wild type, *daf-7* mutants and *daf-7;tdc-1* double mutants, respectively) or the *daf-1(m40)* (**I,** n = 5, 4 and 5 assays for wild type, *daf-1* mutants and *daf-1;tdc-1* double mutants, respectively) mutant animals suppresses the delayed-decision phenotype in either of the mutant animals. Double mutants were compared with the respective single mutants using student’s *t* test. Each bar graph reports the average percentage of worms outside the lawn 15 minutes after the start of the assay. Mean ± SEM, ** p ≤ 0.01, *** p ≤ 0.001, n.s.; not significant.

The *C*. *elegans* TGF-β/DAF-7 regulates several physiological processes through the conserved type I and type II TGF-β receptor, DAF-1 and DAF-4, respectively [77, 78]. DAF-7 is found in the sensory neurons OLQ, ADE and ASI, all of which are implicated in sensing bacteria [22, 70, 71, 79, 80]. DAF-7 produced by ASI regulates the satiety-induced quiescence, the entry into an alternative developmental stage under the environmental stress, and the modulation of the lifespan by dietary restriction, and responds to the abundance of food [71, 79–82]. The expression of *daf-7* is induced in the sensory neurons ASJ upon exposure to pathogenic bacteria and DAF-7 in ASJ regulates the avoidance of the pathogen through DAF-1 and DAF-4 receptors [70]. In addition, through DAF-1 and DAF-4, DAF-7 regulates metabolism and fat accumulation [72]. Here, we showed that mutating *daf-7* delayed the decision to leave the food lawn when 2-nonanone was present (Fig 3G). To identify the source of the DAF-7 signal regulating multisensory integration, we tested the transgenic animals that selectively expressed a wild-type *daf-7* cDNA in subsets of *daf-7-*expressing neurons in *daf-7* mutant animals for potential rescuing effects. We found that expressing *daf-7* selectively in ASI (*Pstr-3*::*daf-7* [70]) did not rescue the defects in the integrated response; but expressing *daf-7* in ADE (*Pcat-2*::*daf-7*) or OLQ (*Pocr-4*::*daf-7*) sensory neurons using cell-selective promoters [83–85] rescued the delayed leaving phenotype in the *daf-7(e1372)* mutant animals (Fig 3K–3M). In addition, we found that the canonical mutation in the type I and type II TGF-β receptor, *daf-1(m40)*, similarly delayed the decision to leave the OP50 lawn paired with 2-nonanone (Fig 4A). Expressing either the genomic DNA fragment of *daf-1* or the *daf-1* cDNA selectively in the interneurons RIM and RIC (*Pdaf-1*::*daf-1* or *pRIM/RIC*::*daf-1*; [70, 72]) fully rescued the defect in the *daf-1(m40)* mutant animals (Fig 4B and 4C), while expressing *daf-1* in sensory neurons (*Pbbs-1*::*daf-1* or *Posm-6*::*daf-1*; [70]) was not sufficient to rescue (Figs 4D and S3). Together, these results indicate that the TGF-β/DAF-7 signal produced by the ADE or the OLQ sensory neurons acts through the type I TGF-β receptor DAF-1 in RIM and/or RIC neurons to promote repellent-dependent leaving of a food lawn.

To further interrogate the role of the RIM/RIC neurons in multisensory integration, we examined the transgenic animals that expressed a histamine-gated chloride channel in the RIM and RIC neurons under the histamine-treated condition [86] or the transgenic animals that expressed tetanus toxin [58] in RIM and RIC. We found that these transgenic animals were normal in leaving the OP50 lawn when 2-nonanone was present (Fig 4E and 4F). Since neither the *tdc-1(n3419)* mutant animals that lack tyramine and octopamine nor the *tbh-1(n3247)* mutant animals that lack octopamine are defective in their decisions to leave the OP50 lawn paired with 2-nanone (Fig 3), together, our results suggest that RIM/RIC and the release of the neurotransmitter tyramine and octopamine from these neurons may be suppressed during the integrated response to the simultaneously present food lawn and 2-nonanone. To further interrogate the role of tyramine or octopamine signaling in the *daf-7-* and *daf-1*-dependent integrated response, we tested how removing tyramine and/or octopamine affects the delayed food leaving in the *daf-7(e1372) or daf-1(m40)* mutant animals. Interestingly, both of the *daf-1(m40); tbh-1(ok1196)* and the *daf-7(e1372); tbh-1(ok1196)* double mutant animals [72] behaved like the *daf-1(m40)* and the *daf-7(e1372)* single mutants, respectively (Fig 4G). In contrast, the mutation in *tdc-1(ok914)* strongly suppressed the delayed decision phenotype in both *daf-7(e1372)* and *daf-1(m40)* mutant animals (Fig 4H and 4I). While TDC-1 is needed for the production of tyramine and octopamine in both RIM and RIC, TBH-1 is only needed for the biosynthesis of octopamine in RIC [66]. Together, these results show that the TGF-β/DAF-7 regulates the decision between staying on a food lawn versus avoiding a repellent through the canonical signaling pathway and that the DAF-7 signal produced from ADE or OLQ inhibits the tyramine neurotransmission of RIM and/or RIC to promote the decision to leave the food-lawn that is paired with 2-nonanone.

### Different interneurons play opposite roles in multisensory integration

**Fig 5:**
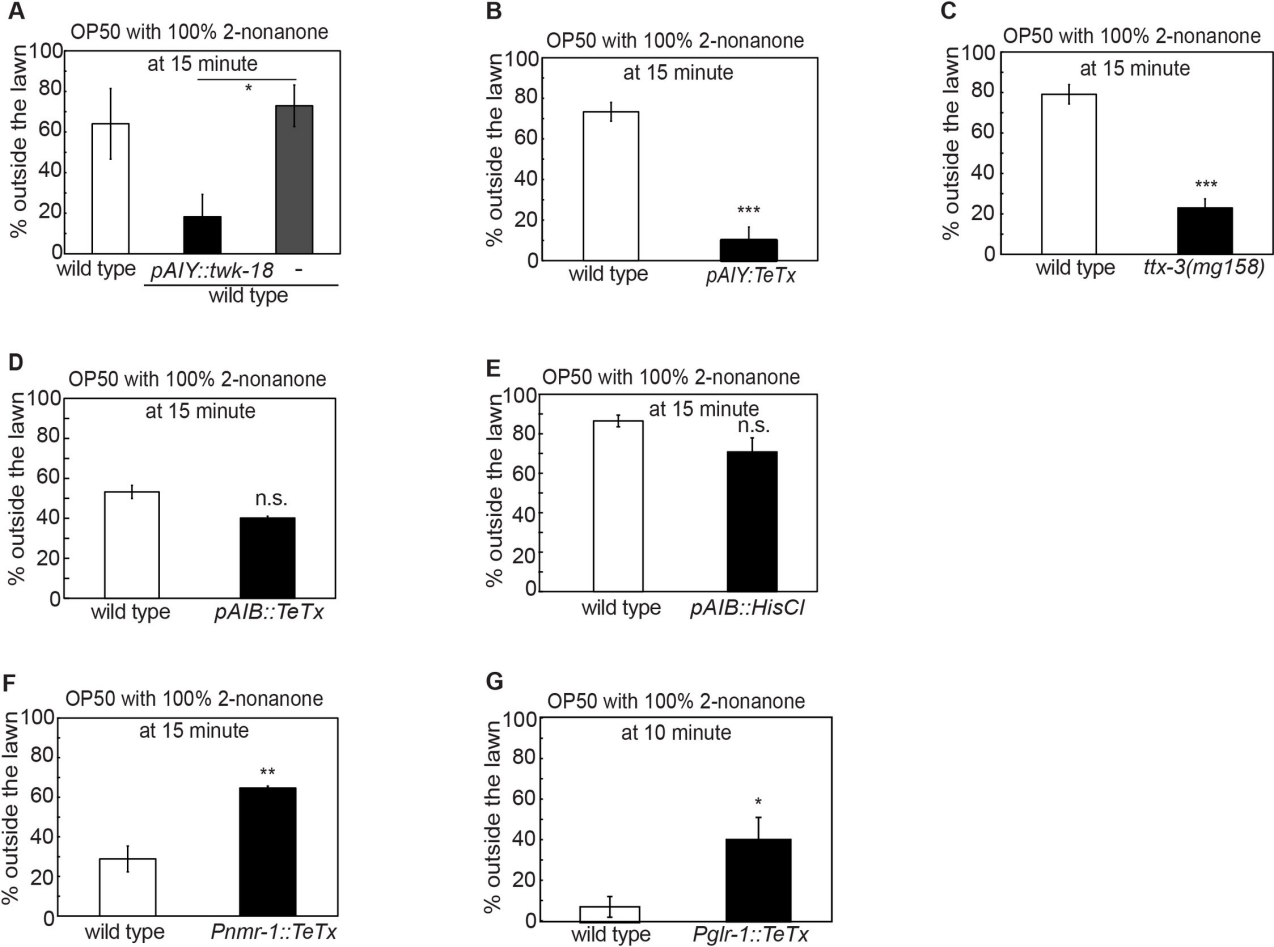
**Downstream circuit that regulates 2-nonanone-dependent food leaving (A-C)** Inhibiting the activity of the AIY interneurons by expressing the gain-of-function isoform of the potassium channel TWK-18 (**A,** *Pttx-3*::*twk-18(gf)*, n = 3 assays each) or by blocking the synaptic outputs of AIY by expressing tetanus toxin (**B,** *Pttx-3*::*TeTx*, n = 4 assays each), or the mutation *ttx-3(mg158)* that generates development defects in AIY (**C,** n = 3 assays each) delays the decision to leave the OP50 lawn paired with 100% 2-nonanone.**(D, E)** Selectively expressing tetanus toxin (**D,** *Pinx-1*::*TeTx*, n = 2 assays each) or the inhibitory HisCl channel (**E,** *Pinx-1*::*HisCl*, n = 3 and 4 assays for wild type and transgenic animals, respectively) in the AIB interneurons does not significantly alter the lawn-leaving decision, when the OP50 lawn is paired with 100% 2-nonanone.**(F, G)** Blocking synaptic outputs from the *nmr-1-*expressing neurons (**F,** *Pnmr-1*::*TeTx*, n = 3 assays each) or the *glr-1-*expressing neurons (**G,** *Pglr-1*::*TeTx*, n = 4 and 3 assays for wild type and transgenic animals, respectively) enhances the 2-nonanone-dependent lawn leaving. Each bar graph reports the average percentage of worms outside the lawn 15 minutes after the start of the assay (**A-F**), unless otherwise specified (**G**). Mean ± SEM, mutants are compared with wild-type animals with student’s *t* test, transgenic animals are compared with non-transgenic siblings or wild type with student’s *t-*test, * p ≤ 0.05, ** p ≤ 0.01, *** p ≤ 0.001, n.s., not significant.

To better characterize the neural circuits underlying multisensory integration, we probed the potential interneurons that regulated the decision between staying on the food lawn versus avoiding 2-nonanone. We focused on the interneurons AIY, AIB, and the command interneurons, all of which regulate locomotion [21, 87]. AIY and AIB are also the major interneurons postsynaptic to the sensory neurons that respond to the bacteria food or the repellent 2-nonanone [39]. To disrupt the function of AIY, we selectively expressed in AIY a gain-of-function isoform of a potassium channel TWK-18 [57] to inhibit the activity of AIY (*Pttx-3*::*twk-18(gf)*) or the tetanus toxin (*Pttx-3*::*TeTx*) to block the synaptic release. We also tested the *ttx-3(mg158)* mutants that fail to develop AIY interneurons [88]. All three mutations delayed the decision to leave the lawn (Fig 5A–5C). However, these manipulations do not disrupt the ability to reach the edge of the food lawn during 2-nonanone-dependent food leaving, to avoid 2-nonanone alone, or to stay on OP50 lawn when no repellent was present (Tables 1–3). In contrast, selectively expressing the tetanus toxin in the AIB interneurons or treating the transgenic animals expressing the histamine-gated chloride channel in AIB with histamine did not significantly alter the decision to leave the OP50 lawn that was paired with 2-nanonone (Fig 5D and 5E). Together, these results indicate that the activity and the synaptic output of the AIY interneurons promote the decision to leave the food lawn paired with 2-nonanone, while AIB are dispensable for the decision-making. Next, we examined transgenic animals that expressed the tetanus toxin with the *nmr-1* promoter or the *glr-1* promoter. The *nmr-1* promoter is expressed in a few command interneurons including AVA, AVB, AVD, AVE and PVC, while the *glr-1* promoter is expressed in several head motor neurons in addition to the *nmr-1-*expressing interneurons [89]. Interestingly, both transgenic lines left the 2-nonanone paired food lawn more than wild type (Fig 5F and 5G). However, these transgenic animals are normal in 2-nonanone avoidance in the absence of food or spontaneous food leaving. They also do not reach the edge of the lawn more rapidly than wild type (Tables 1–3). Together, these results show that different downstream neurons modulate the decision to leave the food lawn paired with a repellent in opposite ways by promoting or inhibiting the decision-making process. These neurons may act as the convergent sites to process multiple sensory signals in order to generate specific behavioral outputs.

### Multisensory integration is regulated by a common set of modulators

**Fig 6:**
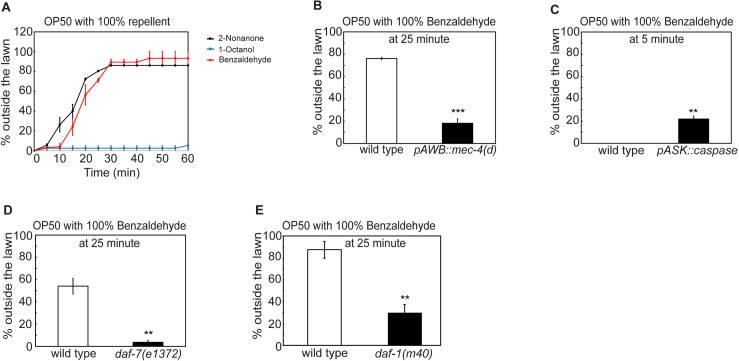
Integrated response to a repellent-paired food lawn is regulated by a common set of factors. **(A)** Wild-type animals also leave the lawn of OP50 paired with 100% benzaldehyde; in contrast, paring an OP50 lawn with 100% 1-octanol does not repel worms (n = 2 assays for each condition).**(B-E)** Genetic ablation of the sensory neurons AWB (**B,** *pAWB*::*mec-4(d)*, n = 3 assays each) or ASK (**C,** *pASK*::*caspase*, n = 3 assays each) or mutating the genetic components of the TGF-β/DAF-7 pathway (**D,** *daf-7(e1372)*, n = 3 assays each; **E,** *daf-1(m40)*, n = 4 assays each) alters the decision to leave the benzaldehyde-paired OP50 lawn. Each bar graph reports the average percentage of worms outside the lawn 25 minutes (**B, D, E)** or 5 minutes (**C**) after the start of the assay. Mean ± SEM, mutants or transgenic animals are compared with wild-type animals with Student’s *t* test, ** p ≤ 0.01, *** p ≤ 0.001, n.s., not significant.

**Fig 7:**
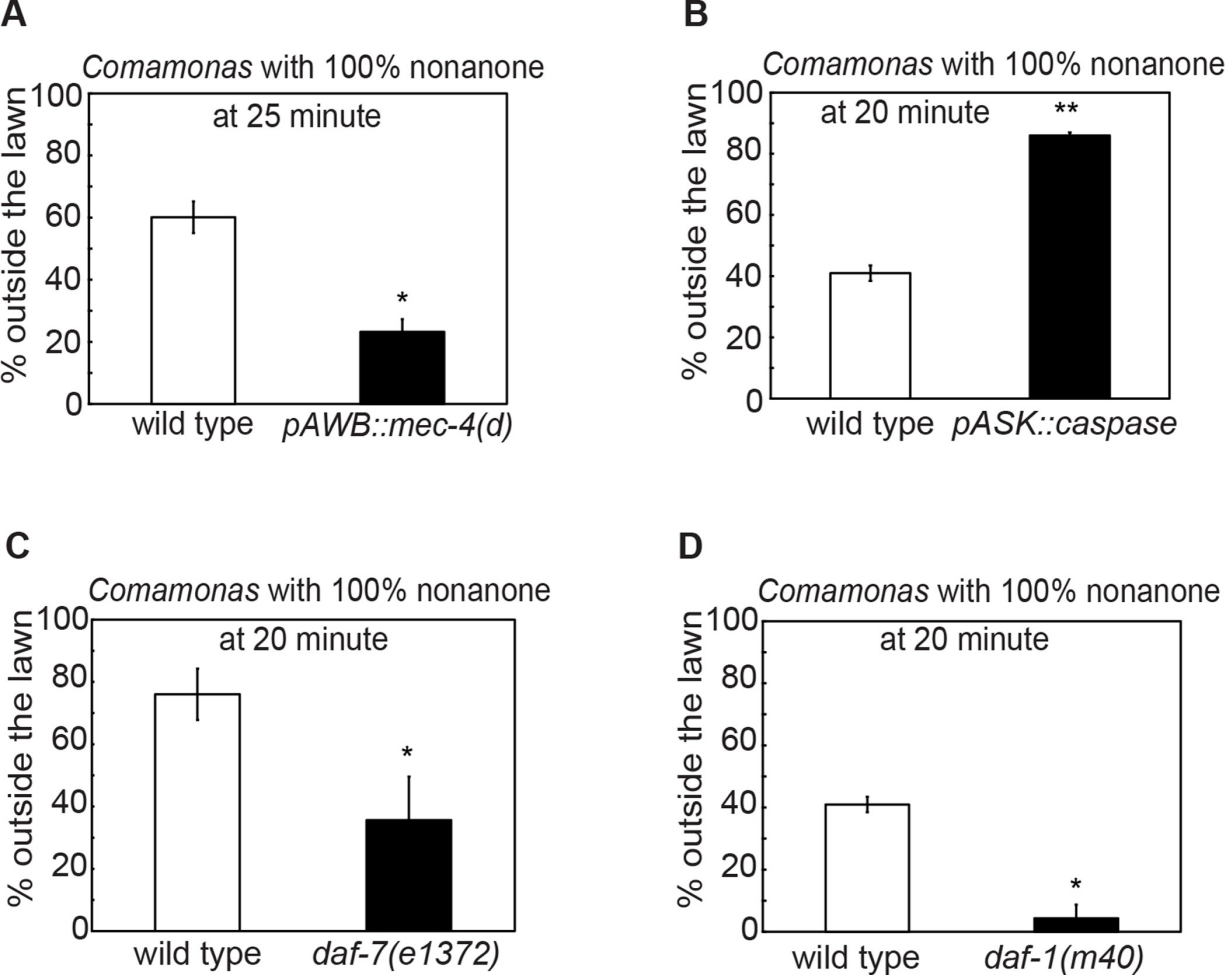
Integrated response to a repellent-paired food lawn requires a common set of factors. **(A-D)** Genetic ablation of the sensory neurons AWB (**A,** *pAWB*::*mec-4(d)*, n = 2 assays each) or ASK (**B,** *pASK*::*caspase*, n = 2 assays each), or mutating the genetic components of the TGF-β/DAF-7 pathway (**C,** *daf-7(e1372)*, n = 4 assays each; **D,** *daf-1(m40)*, n = 2 assays each) alters the decision to leave the 2-nonanone-paired *Comamonas* lawn. Each bar graph reports the average percentage of worms outside the lawn 25 minutes (**A**) or 20 minutes (**B-D**) after the start of the assay. Mean ± SEM, mutants or transgenic animals are compared with wild type with Student’s *t* test, * p ≤ 0.05, ** p ≤ 0.01.

Next, we asked whether the molecular and circuit mechanisms underlying the integrated response to the OP50 food lawn paired with 2-nonanone were shared by the integrated responses to different pairing of attractive foods and repulsive odorants. We paired the OP50 lawn with various repellents, including 100% 1-octanol and 100% benzaldehyde. While benzaldehyde is attractive at low concentrations [23], 100% benzaldehyde strongly repels *C*. *elegans* in a way that is dependent on the function of the sensory neurons AWB [90–92]. We found that a drop of 100% benzaldehyde first repelled the animals to the edge of the OP50 food lawn and then in about 10–15 minutes started to repel the animals off the food lawn (Fig 6A and S2 Movie). This decision to leave depends on the function of the sensory neurons AWB (Fig 6B). Interestingly, 1-octanol failed to stimulate food leaving under our experimental conditions (Fig 6A). We also paired a lawn of *Comamonas sp* with 100% 2-nonanone. *Comamonas* is an attractive food source for *C*. *elegans* [40]. We found that pairing a *Comamonas* bacteria lawn with 100% 2-nonanone repelled *C*. *elegans* off the lawn similarly as the OP50 lawn paired with 2-nonanone (Fig 7A). Interestingly, we found that several modulators, particularly TGF-β/DAF-7, the TGF-β receptor DAF-1, and the sensory neurons ASK, that regulated the integrated response to an OP50 lawn paired with 100% 2-nonanone also similarly regulated the integrated response to OP50 lawn paired with 100% benzaldehyde and the integrated response to the *Comamonas* lawn paired with 100% 2-nonanone (Figs 6 and 7). Together, these results indicate that a common set of modulators and signaling mechanisms regulates the integrated behavioral decisions on whether to leave or stay on an attractive food lawn paired with an odorant repellent. For freely feeding animals, such as *C*. *elegans*, appropriate behavioral responses to food sources paired with other sensory cues are critical for survival, because food can be easily found in close proximity to toxins. It is conceivable that a common set of modulators represent the contexts where the worm needs to evaluate the opposing values provided by a source of nutrients and a potential threat to generate a behavioral decision.

## Discussion

**Fig 8:**
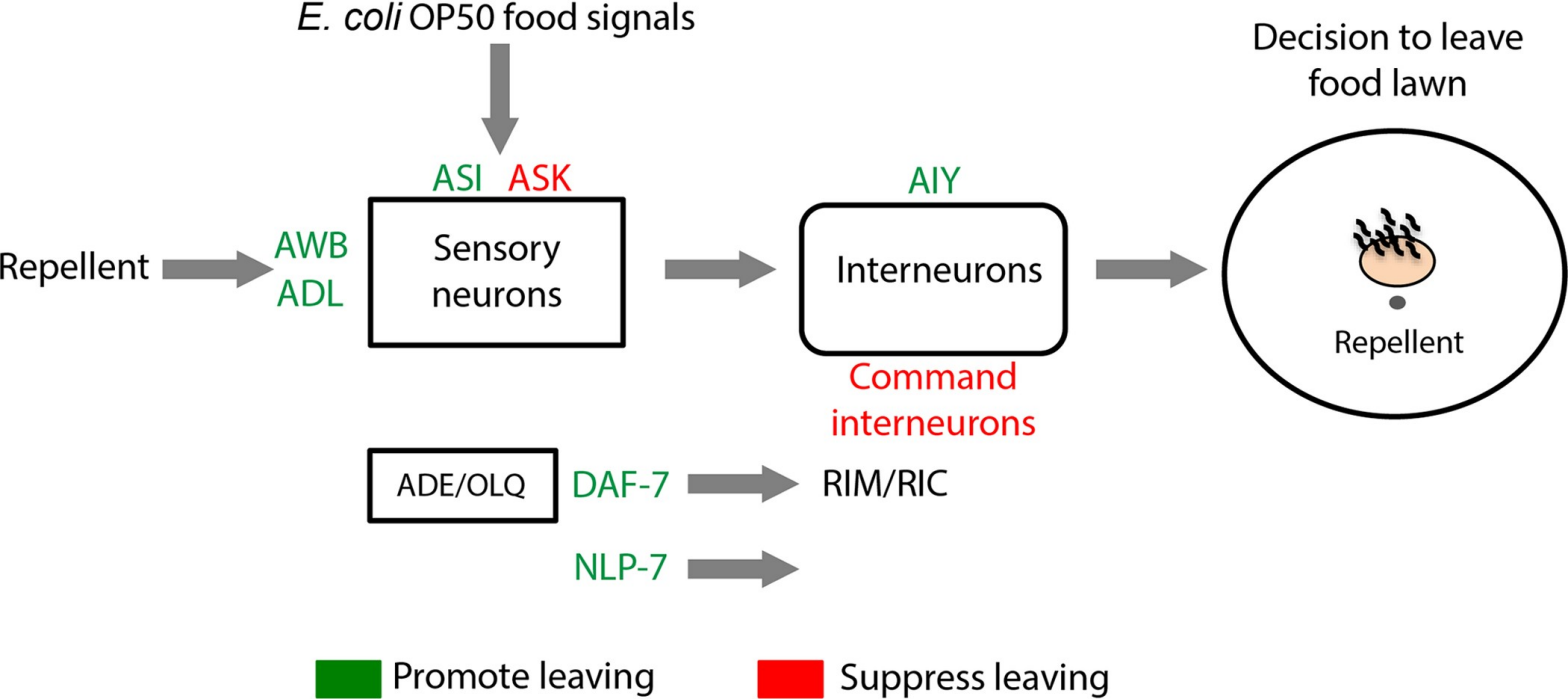
A summary of the identified regulators of a multisensory integration behavior. Summary of the neuronal signaling molecules and neurons that regulate 2-nonanone-dependent food leaving. DAF-7, a TGF-β; NLP-7, a neuropeptide; RIM/RIC, AIY, interneurons; ASK, ASI, AWB, ADL, sensory neurons; ADE and OLQ, sensory neurons that express *daf-7*; command interneurons, AVA, AVD, AVB, PVC neurons. Green and red represent regulators that promote or suppress 2-nonanone-dependent food leaving, respectively.

Many organisms can combine information from multiple simultaneously present sensory cues to generate appropriate behavioral responses [1–8, 31]. By establishing a behavioral paradigm for multisensory integration and characterizing the underlying molecular and cellular mechanisms, we identify the modulators and signaling pathways that regulate a decision to leave a food lawn that is paired with a repulsive odorant (Fig 8).

### Specific sensory neurons regulate multisensory integration

One potential mechanism to regulate a coherent behavioral response to multiple simultaneously present sensory cues is to utilize sensory neurons that are capable of perceiving some or all of the cues. These types of sensory responses can involve either the activation or the inhibition of certain sensory neurons that detect distinct stimuli. Worms are capable of sensing both food signals and a range of repulsive cues ([31] and the references therein) using a variety of sensory neurons. Here, we confirm the requirement of the AWB sensory neurons that are known to sense repellents, including 2-nonanone and 100% benzaldehyde [42, 44, 90]. AWB also respond to bacteria food [43, 50]. Previous studies identify the role of AWB in promoting food leaving under malnourished conditions [93], suggesting the involvement of AWB in integrating the nutritional state with the food signals. Thus, AWB may regulate the integrated response by simultaneously processing food smells and repulsive odorants.

Interestingly, we also uncover a critical and specific role of three sensory neurons, ASK, ADL and ASI, in modulating the decision to leave a food paired with a repulsive odorant. Previous studies show that ASK and ASI sensory neurons respond to *E*. *coli* OP50 [50, 94]. Both ASI and ASK are involved in evaluating the properties of the food in the environment [49]. ASI also mediate the balance between food intake and fat storage, as well as experience-dependent changes in food response [36, 72, 79–81, 95]. ASK regulate the responses to the pheromones and mediate food leaving in animals that are significantly food-deprived [62, 63, 93]. Thus, it is possible that ASI and ASK neurons sense the olfactory signal of food and/or the satiety signal to regulate the multisensory integration. The ADL sensory neurons have been shown to regulate the responses to the repellent octanol, the pheromones and the preference for certain food odors [42, 63, 76, 96]. Therefore, it is conceivable that ADL regulate the multisensory integration by mediating the response to the repulsive odorants.

### Neuropeptides and growth factors modulate multisensory integration

How neuromodulators regulate a behavioral decision that integrates cues of opposing values is not well understood. Previous studies in *C*. *elegans* have implicated neuropeptides and growth factors in the context-dependent modulation of several sensorimotor responses [3, 33, 76, 95, 97, 98]. Here, we show that the NLP-7 peptide and TGF-β/DAF-7 modulate the decision to leave a food lawn paired with a repulsive odorant. *nlp-7* is expressed in several amphidial sensory neurons that respond to contextual cues and NLP-7 delays the acute avoidance of a noxious stimulus, 1-octanol [73–76, 99]. This effect is in contrast with that of mutating *nlp-7* in the integrated behavioral response, where NLP-7 promotes the decision to leave the food in order to avoid the repulsive odorant. These results together characterize distinct functions of the NLP-7 neuropeptide in regulating multisensory integration versus context-dependent avoidance of noxious stimuli.

The TGF-β/DAF-7 pathway regulates multiple behavioral and physiological events, including dauer formation, food intake, fat storage, as well as avoidance of pathogenic bacteria after prolonged exposure. The functions of DAF-7 in these physiological events depend on its expression in the sensory neurons ASI and/or ASJ [70–72]. Here, we show that DAF-7 promotes the decision to leave the food lawn paired with a repulsive odorant via its expression in either the ADE or the OLQ sensory neurons. Our results are the first to characterize the function of *daf-7* produced by ADE or OLQ. ADE produce dopamine [100]. However, we did not see any phenotype in the *cat-2* mutants that were defective in dopamine synthesis (Fig 3 and Table 4), suggesting that the function of ADE in regulating the integrated response to food and repellent is independent of dopamine. These results distinguish the role of *daf-7* in the aforementioned food-associated behaviors from its function in the 2-nonanone-dependent lawn-leaving. Although DAF-7 likely functions in a way that is independent of synaptic connections, the cell-specific function of DAF-7 in different behaviors may result from the functions of different *daf-7-*expressing sensory neurons in detecting and responding to different environmental conditions. Previous studies show that the *daf-7* expression level in ASJ increases rapidly after incubation on the pathogenic bacteria PA14 [70]. It will be informative to examine whether the *daf-7* expression similarly changes during the first several-minute simultaneous exposure to the food and 2-nonanone and whether the potential change causally links with the lawn-leaving behavior. ADE and OLQ have been previously implicated in mechanosensation [22, 30, 101]. Thus, we propose that ADE and OLQ regulate the integrated response to a food lawn paired with a repellent by representing the mechanical stimulus that a worm senses from the food lawn. Further studies to characterize the function of these neurons using *in vivo* calcium imaging and by manipulating their activity or the signal release and testing the resulting behavioral effect will be informative for understanding their roles in regulating multisensory integration.

We further show that the canonical TGF-β receptor DAF-1 acts in the interneurons RIM and RIC to regulate the decision to leave the food lawn paired with a repulsive odorant by inhibiting the tyramine signaling from these interneurons. This regulatory mechanism of DAF-7 is reminiscent of that in feeding, where DAF-7 promotes the pumping rate by inhibiting the output from the RIM and/or RIC neurons [72]. The RIM and RIC neurons have been previously implicated in various sensorimotor responses, as well as the context-dependent locomotory and feeding behaviors [66, 72, 86, 99, 102]. Our results further reveal RIM/RIC as one of the central sites where different sensory signals converge to generate appropriate behavioral outputs. The members in the large TGF-β family have been implicated in various neuronal functions, including learning and memory, synaptic plasticity, synaptogenesis, dendritic development, and regulation of the function of the neural-muscular junctions [103–106]. Defects in the TGF-β pathways have been implicated in the pathology of neurological disorders, such as schizophrenia, depression, anxiety and Alzheimer’s disease [107–109]. Our work reveals a new role for TGF-β signals in regulating decision-making, when sensory cues of opposing valance are simultaneously present.

Interestingly, in contrast to the critical role of the NLP-7 peptide and the DAF-7 in the multisensory integration, none of the mutants that lack the major neurotransmitters is defective in the 2-nonanone-dependent lawn-leaving. Previously, several food-associated behaviors have been studied in *C*. *elegans*, some of which are regulated by the neurotransmitters. For example, the worm avoids octanol within several seconds upon the exposure to the repellent. The presence of food or serotonin modulates the latency of the response [96]. In addition, the worm spontaneously leaves a food lawn at a very low frequency (< 0.05 leaving event/min) and the rate of leaving is modulated by tyramine and/or octopamine [41]. In our assay, the majority of the worms leave the lawn several minutes after the addition of a repellent to the side of the lawn. The dynamics and the time scale of this repellent-dependent lawn-leaving are different from the food-modulated avoidance of octanol or the spontaneous lawn-leaving. The behavior in this study is also different from the lawn-leaving behavior that is driven by the depletion of the lawn, which occurs over a course of several hours [48], or from leaving a lawn of pathogenic bacteria, in which the worms start to leave the lawn after feeding on the lawn for a few hours due to the pathogenesis of the bacteria [70]. Thus, it is conceivable that the lawn-leaving behavior that our assay analyzes is regulated by mechanisms that are different from those important for the other food-associated behaviors.

### Specific interneurons modulate 2-nonanone-dependent food leaving in *C*. *elegans*

The ability to integrate multiple types of sensory stimuli requires not only the responses across peripheral sensory areas, but also the signal processing in downstream network of interneurons [1, 3, 5–8, 110]. In *C*. *elegans*, a number of sensorimotor responses are modulated by specific contexts via the functions of several interneurons [33, 97, 99, 111]. However, how interneurons mediate decision-making during multisensory behavior is not fully characterized. Here, we find that the AIY interneurons play a modulatory role in 2-nonanone-dependent food leaving. The AIY interneurons receive synaptic inputs from the sensory neurons that detect olfactory, gustatory and thermal information. Previous studies implicate AIY in integrating simultaneously present aversive and attractive cues in olfactory plasticity and in food and serotonin-dependent modulation of sensorimotor responses [33, 111–113]. We propose that AIY may act as an integrating site that receives and processes signals from the food and the repellent 2-nonanone during multisensory integration. Future studies that examine the activity of AIY in response to the simultaneous stimulation of the repellent and the food, as well as to each stimulus alone will further reveal the role of AIY in the multisensory integration. Our study also implicates the *glr-1-* and *nmr-1-*expressing neurons in regulating the repellent-dependent food leaving. It is conceivable that some of these command interneurons or head motor neurons may serve as the downstream-modulated targets for the integrated behavioral response.

## Methods

### Strains

*C*. *elegans* strains were cultivated under the standard conditions [114]. Hermaphrodites were used in this study. The strains that were used in the study include: PR680 *che-1(p680)I*, CX14394 *npr-5(ok1583)V*, MT15434 *tph-1(mg280)II*, CB1112 *cat-2(e1112)II*, *MT9455 tbh-1(n3247)X*, *RB1161 tbh-1(ok1196)X*, *RB993 tdc-1(ok914)II*, *MT13113 tdc-1(n3419)II*, DR40 *daf-1(m40)IV*, PR691 *tax-2(p691)I*, *PR671 tax-2(p671)I*, RB859 *daf-22(ok693)II*, *OH8 ttx-3(mg158)X*, *MT150 egl-3(n150)V*, CX4 *odr-7(ky4)X*, *CX03572 nlp-9(tm3579)V*, ZC2685 *npr-2(ok419)IV*, VC48 *kpc-1(gk8)I*, RB1341 *nlp-1(ok1470)X*, RB1289 *npr-18(ok1388)X*, CB1372 *daf-7(e1372)III*, *ZC2673 gcy-33(ok232)V*, *SM2322 daf-7(ok3125)III*, AX1295 *gcy-35(ok769)I*, QZ81 *ins-6(tm2416)II*, QZ126 *ins-7(tm2001)IV*, FX02105 *nlp-24(tm2105)V*, *RB1902 flp-19(ok2460)x*, CX10 *osm-9(ky10)IV*, FX02984 *nlp-7(tm2984)X*, RB1161 *tbh-1(ok1196)X*, RB993 *tdc-1(ok914)II*, *KQ361 tdc-1(ok914)II; daf-7(e1372)III*, *KQ363 tdc-1(ok914)II; daf-1(m40)IV*, *KQ364 daf-1(m40)IV; tbh-1(ok1196)X*, *KQ362 daf-7(e1372)III; tbh-1(ok1196)X*, *ZC1952 yxIs25[Pttx-3*::*TeTx*::*mCherry; Punc-122*::*gfp]*, KQ280 *daf-1(m40)IV; ftEx98[Pdaf-1*::*daf-1*::*gfp; Podr-1*::*dsRed]*, KQ380 *daf-1(m40)IV; ftEx205*[*Ptdc-1*::*daf-1*::*gfp*; *Podr-1*::*dsRed*], KQ252 *daf-1(m40)IV; ftEx70*[*Pbbs-1*::*daf-1*::*gfp; Podr-1*::*dsRed*], ZD736 *daf-7(ok3125)III;qdEx44[Pstr-3p*::*daf-7; Pges-1*::*gfp]*, ZD729 *daf-7(ok3125)III;qdEx37[Pdaf-7*::*daf-7; Pges-1*::*gfp]*, PY7502 *yxIs34[Pceh-36∇*::*TU813; Pceh-36∇*::*TU814*; *Psrtx-1*::*gfp*; *Punc-122*::*dsRed]*, ZC2393 *yxEx1248 [Pttx-3*::*twk-18(gf)*::*mCherry; Punc-122*::*RFP]*, CX14848 *kyEx4866[Pinx-1*::*HisCl1*::*SL2mCherry; Punc-122*::*dsRed*], CX16040 *kyEx5464[Ptdc-1*::*HisCl1*::*SL2mCherry]*, ZC1451 *yxEx699[Pnmr-1*::*TeTx*::*mCherry; Punc-122*::*dsRED];* QS4 *qrIs2[Psra-9*::*mCasp1; Psra-9*::*gfp; Pelt-2*::*gfp]*, PS6025 *qrIs2[Psra-9*::*mCasp1; Psra-9*::*gfp; Pelt-2*::*gfp]*; ZC1552 *yxEx749[Pglr-1*::*TeTx*::*mCherry; Punc-122*::*gfp]*, PY7505 *oyls84[Pgpa-4*::*TU813; Pgcy-27*::*TU814; Pgcy-27*::*gfp; Punc-122*::*dsRed]*, CX3830 *kyIs102V; kyIs104[Pstr-1*::*mec-4(d); Pstr-1*::*gfp];* CX14637 *kyEx4779[Pinx-1*::*TeTx*::*mCherry; Punc-122*::*gfp]*, CX14993 *kyEx4962[Ptdc-1*::*TeTx*::*mCherry]*, AX2051 *Ex[Pgcy-33*::*egl-1; Punc-122*::*dsRed]*, *CX12330 Ex[Psre-1*::*TeTx*::*mCherry; Punc-122*:*RFP]*, *CX7102 lin-15B(n765)X; qaIs2241[Pgcy-36*::*egl-1; Pgcy-35*::*gfp; lin-15(+)]*, *ZC2752 nlp-7(tm2984)X; yxEx1420[Pnlp-7*::*nlp-7; Punc-122*::*gfp]*, *ZC2731 daf-7(e1372)III; yxEx1409[Pcat-2*::*daf-7; Punc-122*::*gfp]; ZC2734 daf-7(e1372)III*, *yxEx1412[Pocr-4*::*daf-7; Punc-122*::*gfp]*

### Behavioral assay for multisensory integration

On a 5 cm-diameter NGM (Nematode Growth Medium, 2.5g/L Bacto Peptone, 3.0g/L NaCl, 1mM CaCl2, 1mM MgSO4, 25mM KPO4 pH6.0) plate, 15–25 young adult worms were placed on a small 1 cm-diameter round-shaped bacteria lawn to acclimatize for 1 hour. The lawn was made by putting a drop of freshly prepared *E*. *coli* OP50 culture on the NGM plate and letting the plate stand on the bench for 2 hours. Next, a drop of 1 μl 2-nonanone (Sigma Aldrich, Cat # 821-55-6), either 10% (v/v in 100% ethanol) or 100%, was placed on the right-hand side of the lawn and 1–3 mm away from the lawn. The number of worms on the lawn was counted every 5 minutes for a total of 60 minutes, and the percentage of worms outside the lawn was calculated (Fig 1A and 1B). In some assays, 1 μl of 100% benzaldehyde (Sigma Aldrich, Cat # 100-52-7) was used, instead of 2-nonanone. The OP50 culture was prepared freshly each day by culturing at 27°C for 12–15 hours in NGM medium. For assays using *Comamonas sp* for the food lawn, the experiments were performed in the same way, except that the bacteria strain was cultured with Luria Broth. To determine the time taken to reach the edge of the food lawn, the food lawn was divided into 5 columns with each being 2 mm wide (S1 Fig). The time taken for 90% of the worms to crawl into the column furthest away from the repellent was recorded. The percentage of worms that leave the repellent-paired food lawn quantified on different days may vary. The main source of the variability is likely the variability of the food-lawn, which is made by the bacteria culture freshly prepared every day. Although the bacteria culture is prepared using the same method each time, we may not be able to control all aspects of the bacteria growth, which can generate difference in the assay conditions, such as the thickness of the lawn and the amount of the bacterially derived metabolites present in the lawn. These variables may affect the dynamics of the behavior. However, the effects of these variations were minimized, because we always compared mutants with wild type tested in parallel, and compared transgenic animals with non-transgenic siblings or wild-type animals tested in parallel on the same days. The bar graphs in the figures report the percentage of worms outside the lawn 15 minutes after the start of the assay, unless otherwise noted. We used this time point, because it was often when a significant difference was first observed.

### Transgenes and transgenic animals

To generate a *nlp-7* genomic rescue fragment, a 4.7 kb PCR product was amplified from genomic DNA that included 2.5 kb 5’ upstream sequence, the *nlp-7* coding region, and 1 kb 3’ downstream sequence (NLP-7F: 5’-CATGTTTTTGATCATTTTCGAAC-‘3 and NLP-7R3’UTR: 5’-AATATCGTATGCCAACTTGAAC-‘3). The *nlp-7* genomic PCR product was injected into the *nlp-7(tm2984)* animals. To generate the construct expressing a wild-type *daf-7* cDNA in the OLQ or ADE sensory neurons, the *daf-7* cDNA was amplified from PJM016 (Gift from Dr. Dennis Kim and Dr. Joshua Meisel [70]). The *daf-7* cDNA product was cloned into a gateway destination vector that contained an *unc-54* 3’UTR using the Nhe-1 and Kpn-1 sites. Both the promoter regions of *ocr-4* (4.0 kb promoter for expression in OLQ) and *cat-2* (1.1 kb promoter for expression in ADE) were amplified from genomic DNA (CAT-2F: CTAGCAGGCCCAATCTTTTCTG and CAT-2R: TCCTCTTCCAATTTTTCAAGGGGT/OCR-4F: 5’-TTCTAATATTGCTCCATCAAC-‘3 and OCR-4R: 5’-TAATACAAGTTAGATTCAGAGAATA-‘3) and cloned into the entry-TOPO vector PCR8 (Invitrogen). The expression clones, *Pcat-2*::*daf-7* and *Pocr-4*::*daf-7*, were generated using LR recombination reactions (Invitrogen). Each transgene was injected at 30–50 ng/μl with the co-injection marker as previously described [115].

### Lawn-leaving assay

Lawn-leaving assay was performed and analyzed similarly as the assay for multisensory integration, except that no repulsive chemical was present. Briefly, animals were placed on a 1 cm-diameter round-shaped bacteria lawn of OP50 and left for 10 minutes to acclimatize before examining food leaving over a period of one hour by counting the number of worms that were present on the food lawn every 5 minutes for a total of 60 minutes.

### 2-nonanone avoidance assay

To examine the avoidance of 2-nonanone, chemotaxis assays were performed essentially as previously described [42]. Briefly, animals were placed in the center of a square plate with a side of ~ 9 cm that was divided into sectors A—F and 2 drops of 1 μl of 2-nonanone was added to one side and 2 drops of 1 μl ethanol was added to the opposite side of the plate as control. Approximately 100 worms were used in each assay. Chemotactic avoidance was analyzed by counting the number of worms in the sectors A-B, C-D and E-F with E-F being furthest away from the 2-nonanone point sources (S1 Fig). The avoidance index was calculated as the number of animals in sectors A and B minus the number of animals in the sectors E and F and normalized with the total number of animals in all 6 sectors on plate.

## Supporting information

S1 Fig Schematics of assays. **(A)** Assay to measure the time taken to reach the edge of the food lawn (Methods).
**(B)** Chemotaxis assay for avoidance of 100% 2-nonanone (Methods).
(PNG)

S2 Fig Additional alleles of *tdc-1* and *tbh-1* mutants are also wild-type for 2-nonanone-dependent food leaving. Each bar graph shows the percentage of animals outside the food lawn 15 minutes after the start of the assay, mutants are compared with wild type tested in parallel with Student’s *t* test, n = 3 assays each; mean ± SEM, n.s., not significant.
(PNG)

S3 Fig Expressing the *daf-1* cDNA in sensory neurons with *osm-6* promoter does not rescue the delayed-decision phenotype in the *daf-1(m40)* mutants. The transgenic animals (n = 3 assays) are compared with non-transgenic siblings (n = 4 assays) with Student’s *t* test, wild type = 3 assays; bar graph shows the percentage of worms outside of lawn 15 minutes after the start of the assay, mean ± SEM, n.s., not significant.
(PNG)

S1 Movie Wild-type worms performing food leaving on an *E*. *coli* OP50 lawn paired with 100% 2-nonanone. (MP4)

S2 Movie Wild-type worms performing food leaving on an *E*. *coli* OP50 food lawn paired with 100% benzaldehyde. (MP4)

S1 Dataset Data for the graphs in Fig 1. (XLSX)

S2 Dataset Data for the graphs in Fig 2. (XLSX)

S3 Dataset Data for the graphs in Fig 3. (XLSX)

S4 Dataset Data for the graphs in Fig 4. (XLSX)

S5 Dataset Data for the graphs in Fig 5. (XLSX)

S6 Dataset Data for the graphs in Fig 6. (XLSX)

S7 Dataset Data for the graphs in Fig 7. (XLSX)

S8 Dataset Data for the graphs in S2 and S3 Figs. (XLSX)
