# Supplementary figures and images for: Molecular and cellular modulators for multisensory integration in C. elegans
Source: PLoS Genet. 2019 Mar 8;15(3):e1007706. doi: 10.1371/journal.pgen.1007706 (PMC6426271; doi:10.1371/journal.pgen.1007706)

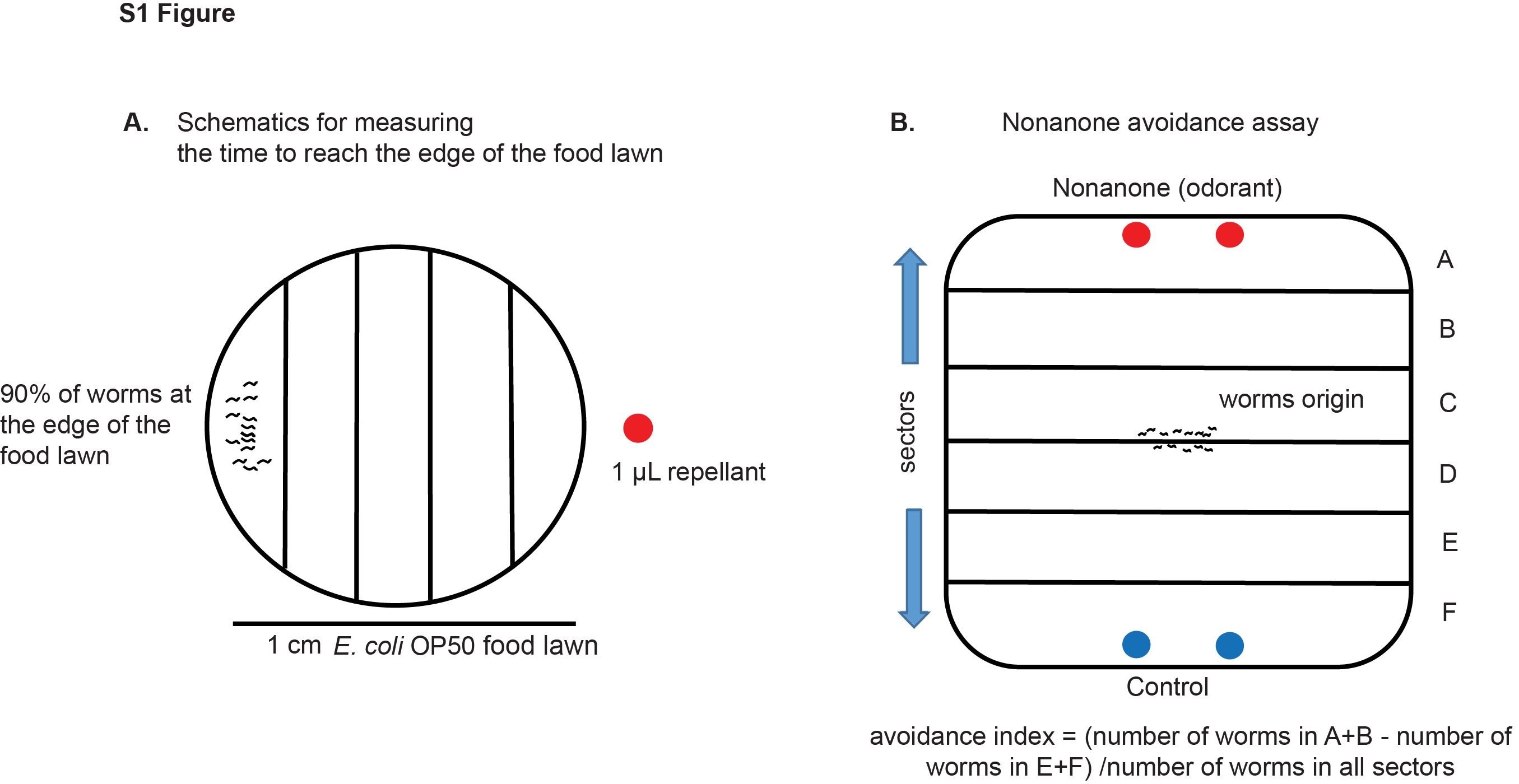

Supplement: S1 Fig — (A) Assay to measure the time taken to reach the edge of the food lawn (Methods). (B) Chemotaxis assay for avoidance of 100% 2-nonanone (Methods). (PNG) [file pgen.1007706.s001.png]

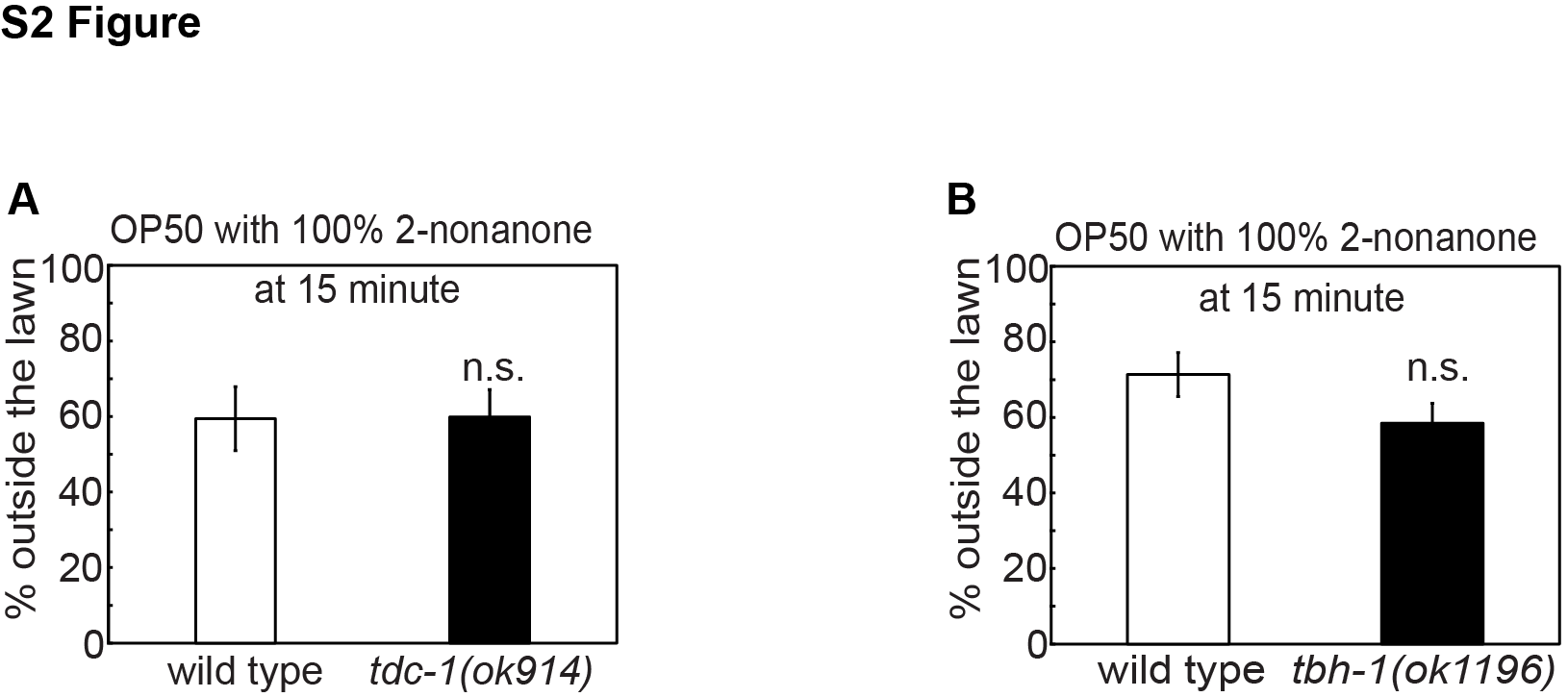

Supplement: S2 Fig — Each bar graph shows the percentage of animals outside the food lawn 15 minutes after the start of the assay, mutants are compared with wild type tested in parallel with Student’s t test, n = 3 assays each; mean ± SEM, n.s., not significant. (PNG) [file pgen.1007706.s002.png]

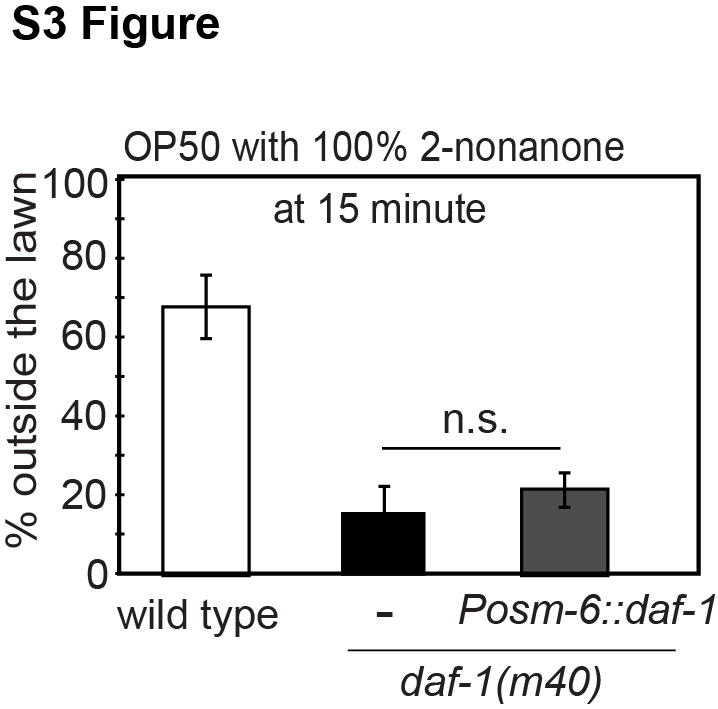

Supplement: S3 Fig — The transgenic animals (n = 3 assays) are compared with non-transgenic siblings (n = 4 assays) with Student’s t test, wild type = 3 assays; bar graph shows the percentage of worms outside of lawn 15 minutes after the start of the assay, mean ± SEM, n.s., not significant. (PNG) [file pgen.1007706.s003.png]
